# Mir‐27a‐3p attenuates bronchiolitis obliterans in vivo via the regulation of dendritic cells’ maturation and the suppression of myofibroblasts’ differentiation

**DOI:** 10.1002/ctm2.140

**Published:** 2020-08-12

**Authors:** Ming Dong, Xin Wang, Tong Li, Jing Wang, Yunwei Yang, Yi Liu, Yaqing Jing, Honglin Zhao, Jun Chen

**Affiliations:** ^1^ Department of Lung Cancer Surgery Tianjin Medical University General Hospital Tianjin P. R. China; ^2^ Department of Pediatric Surgery Tianjin Children's Hospital Tianjin P. R. China; ^3^ Tianjin Key Laboratory of Lung Cancer Metastasis and Tumor Microenvironment Tianjin Lung Cancer Institute Tianjin Medical University General Hospital Tianjin P. R. China; ^4^ School of Basic Medical Sciences Tianjin Medical University Tianjin P. R. China

**Keywords:** bronchiolitis obliterans, dendritic cells, lung transplantation, mir‐27a‐3p

## Abstract

Bronchiolitis obliterans (BO), is a chronic rejection phenotype characterized by chronic small airway fibrous obliteration, hinders the patients who suffer from lung transplanting for surviving longer. Cell‐based therapies using dendritic cells (DCs) and T regulatory cells (Tregs) have been developed to regulate allograft rejection, and to induce and maintain immune tolerance. In the present study, the effects of mir‐27a‐3p on regulating DCs as well as resulting effects on BO attenuation have been investigated. According to our reporter assays, the potential targets of mir‐27a‐3p were Smad2, sprouty2, and Smad4, respectively. Furthermore, sprouty2 inhibition by mir‐27‐3p indirectly activated extracellular regulated protein kinases (ERK) and increased IL‐10 production in DCs. This led to a positive feedback loop that maintained the immature state of DCs via IL‐10/JAK/STAT3 pathway, and caused an increase in Foxp3^+^CD4^+^ T cells amount as well as TGF‐β level. Furthermore, mir‐27a‐3p regulated TGF‐β function, inhibited TGF‐β/Smad pathway, and suppressed myofibroblast differentiation through influencing the function of Smad2 and Smad4. In short, the study indicated the effect of mir‐27a‐3p on suppressing DC maturation, which implicated the potential clinical application in treating postlung transplant BO.

## Graphical abstract

1. Our study confirms the regulatory role of mir‐27a‐3p on DCs and suggests its potential clinical application to treat postlung transplant BO.
2. Mir‐27a‐3p regulates DC maturation by targeting sprouty2 that indirectly activates ERK and promotes IL‐10 through the IL‐10/JAK1/STAT3 protein signaling.
3. Mir‐27a‐3p also regulated TGF‐β function, inhibited the TGF‐β/Smad pathway, and suppressed myofibroblast differentiation by targeting Smad2 and Smad4.
4. We confirmed the regulatory role of mir‐27a‐3p in DCs, providing a feasible clinically applicable strategy for BO treatment after lung transplantation.

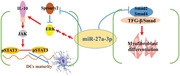

## 1 INTRODUCTION

Lung transplantation (LT) is one of viable life‐saving treatment strategies for terminal‐stage lung disease.^1^ Even so, the 5‐year survival rate is still not significant (about 50%).^2^ Bronchiolitis obliterans syndrome (BOS) is a type of chronic small airway fibrous obliteration and is known as bronchiolitis obliterans (BO).^3^ It is a phenotype of chronic rejection that mainly hinders long‐term survival after LT.^4^ Many immunosuppressive drugs and novel therapies have been developed in the last decade to prevent immunological rejection. However, BO still poses a great problem that has proved difficult to solve.

The exact mechanisms underlying BO pathogenesis remain to be elucidated. Many factors, including immune imbalance,^5^ cytokine response,^6^ epithelial‐mesenchymal transition (EMT),^7^ and myofibroblast differentiation,^8^ contribute to BO. Dendritic cells (DCs)‐ and T regulatory cells (Tregs)‐based therapies have been widely developed to prevent allograft rejection through inducing and maintaining immune tolerance.^9^ DCs play a pivotal role in motivating transplanted organs rejection via activated (effector) T cells and are professional antigen‐presenting cells that elicit an immune response.^10^ Recently, it has been suggested that DCs regulate immune tolerance by inhibiting T cells or inducing Treg cells.^11^ In a series of clinical investigation, the safety of the application of tolerogenic DCs has been proved for autoimmune disorders administration.^12^ The adoptive transfer of tolerogenic DCs exerts antigen‐specific immunosuppressive therapeutic effects that attenuate allograft rejection^13^ with no affection of the salutary immune response. DCs can be derived from DC‐committed precursors (pre‐DCs) in the bone marrow (BM), which contain several subsets based on their phenotypic and functional difference. The main function of DCs includes antigen acquiring, processing, and presentation, which activate priming of T‐cells’ response to an “enemy.”^14^ Additionally, another key role of DCs is to establish self‐tolerance.^15^ Generally, with high expression of MHC II, CD80, and CD86,^16^ mature DCs can induce antidonor immune responses, whereas immature, tolerogenic DCs that express low costimulatory molecules induce immune tolerance.^17^

Tolerogenic DC functions are controlled through the regulation of steady‐state DCs and inhibitory checkpoint molecules that are crucial for the maintenance of immune homeostasis.^18^ Therefore, a tolerogenic DC state is essential for inducing and maintaining immune tolerance.^19^ Moreover, many factors induce DC maturation through different signaling pathways. In the past, it has been demonstrated that the IL‐10/Jak1/STAT3 signaling act as essential roles as negativity adaptor in DC maturity in rat model allograft preservation.^20^ Generated by almost all kinds of leukocytes, including DCs, IL‐10 acts as a major immunosuppressive and anti‐inflammatory cytokine in immunoregulatory processes.^21^ Activation of STAT3 in DCs increases IL‐10 production, acquisition of tolerogenic features, and Treg generation.^22^ IL‐10 has also been suggested to induce a positive feedback circulation; IL‐10 presentation activates Jak1/STAT3, which in turn results in IL‐10 overexpression. Consequently, this inhibits DC maturation and maintains the tolerogenic DCs steady state. MicroRNAs (miRNAs), a kind of small noncoding RNAs containing 20‐25 nucleotides, are of great value in controlling posttranscriptional gene expression.^23^ In our previous study, miR‐27a‐3p downregulation brought about the development of BO through Smad3‐mediated EMT.^7^ However, we did not explore the relationship on miR‐27a‐3p as well as immune regulation. The overexpression of MiR‐27a inhibited the ERK inhibitor, sprouty2, and consequently resulted in overactive of the ERK‐related cell signaling as well as increase of production of IL‐10. In the present study, we illustrated potential role of miR‐27a‐3p on DC maturation, immune regulation, and BO.

## 2 MATERIALS AND METHODS

### 2.1 Cell extraction and cell culture

The extraction of bone marrow cells was operated under germfree conditions from BALB/c mice. The cells were cultured in Dulbecco's modified Eagle medium (DMEM) containing 20 ng/mL of recombinant murine GM‐CSF and10 ng/mL recombinant murine IL‐4 (R&D Systems, Minneapolis, MN) at 37°C with 5% CO_2_.^24^ Next, nonadherent cells were discarded every 2 days, and further incubated with above‐mentioned DMEM complete medium. The NIN‐3T3 cell line was purchased from ATCC (Manassas, VA), and was cultured in RPMI‐1640 medium containing 10% fetal bovine serum (Sijiqing Co. Ltd, Hangzhou, Zhejiang, China) at 37°C in a humidified atmosphere containing 5% CO_2_.

### 2.2 Animals

The adult, pathogen‐free, and female C57BL/6 (H‐2b) mice and Balb/c (H‐2d) mice (8‐10 weeks, 20‐24 g) were obtained from Tianjin Medical University Experimental Animal Center (Tianjin, China). This study was conducted in conformity with the Principles of Laboratory Animal Care (NIH publication Vol. 25, No. 28 revised 1996) as well as the Tianjin Medical University Animal Care and Use Committee Guidelines. All animal‐related studies were conducted on the basis of the protocol authorized by Tianjin Medical University Institutional Animal Care and Use Committee. In order to minimize the suffering and numbers of animals, all the efforts were made.

### 2.3 Animal experimental protocol

First, both the donor mice and the recipient mice were anesthetized using isoflurane. Subsequently, orthotopic tracheal transplantation was operated as previously described.^25^ In brief, the tracheas from C57BL/6 mice were transplanted to allogeneic Balb/c mice, and donor tracheal grafts were orthotopically implanted. Transplant recipients were administered via lateral tail vein 2 × 10^6^ mimics control DCs and mimics mir‐27a‐3p DCs with or without 100 ng/mL lipopolysaccharide (LPS) for 10 consecutive days. On the 30th day (D30) after transplantation, grafts were harvested for further histological assays. All animal experiments were conducted at the Tianjin Medical University Experimental Animal Center (Tianjin, China).

### 2.4 MiR‐27a‐3p binding predictions

We conducted the analysis on website of TargetScan (http://www.targetscan.org/vert_71/).

### 2.5 Luciferase reporter assay

The recombinant luciferase‐reporter plasmids contained putative miR‐27a‐3p binding orders of Sprouty2, Smad2, and Smad4 3′‐untranslated region (UTR). Luciferase intensity was evaluated by Promega luciferase assay system (Promega, USA). Applying the kit of dual‐luciferase reporter gene (GeneCopoeia, China), secreted alkaline phosphatase and Gaussia luciferase were measured after 48 hours of transfection. Renilla luciferase was adopted as internal control to normalize the transfection efficiency.

### 2.6 Real‐time PCR

We used Trizol (Invitrogen, Carlsbad, CA) to extract total RNAs from different treated cells. The cDNA synthesis was performed with Oligo(dT) or random primers included in the Quantscript RT kit (Tiangen, China). Taqman probes for miR‐27a‐3p were obtained from Applied Biosystems (Foster City, CA). The expression of Sprouty2, Smad2, and Smad 4 was determined by a SYBR Green Master Mix kit (Roche, Switzerland). Expression levels were quantified using the 2‐ΔΔCt method in which GAPDH was an internal control. Primers sequences were as follows:

Sprouty2: 5′‐CGGCAAGTGCAAGTGT‐3′ (sense), 5′‐TCCCATCGCTGACCAT‐3′ (antisense).
Smad2: 5′′‐GCAGGAAGAAAAGTGGTGTGAGA‐3′′ (sense), 5′′‐CAGAGCAAGTGCTTGGTATGGTA‐3′′ (antisense).
Smad4: 5′′‐GCTGTTGTTTTTCACTGTTTCCA‐3′′ (sense), 5′′‐GTTTCACTCTCTCCACCTTGTCT‐3′′ (antisense).
GAPDH: 5′′‐GCTGGCGCTGAGTACGTCGTGGAGT‐3′′ (sense), 5′′‐CACAGTCTTCTGGGTGGCAGTGATGG‐3′′ (antisense).

### 2.7 Western blotting assays

Western blotting assays were conducted according to our previously description.^7^ Briefly, cells were lysed by RIPA and total proteins were isolated. The antibodies of sprouty2, ERK, p‐ERK, JAK1, STAT3 (dilution rate 1:1000), pSTAT3 (dilution rate 1:1000), tubulin, and GAPDH were used as internal control and at the dilution level of 1:1000. All above antibodies were purchased from Affinity Bioreagents (Waltham, MA). The results were analyzed and quantified using an Image J software. The ratio of densitometry values to the corresponding tubulin values was used to indicate the relative protein expression.

### 2.8 Flow cytometry

Specific markers of PBMCs and monocytes were labeled and analyzed by flow cytometry as we previously described.^24^ Briefly, after sterilization with alcohol, spleens were isolated from treated Balb/c mice. Subsequently, the spleen samples were at 70 μm by nylon cells mesh (BD Falcon, MA, USA). A red blood cell separation buffer (R&D Systems) was used to remove red blood cells. An FcR blocking agent (Miltenyi Biotec) was adapted to exclude nonspecific antibody combination in PBMCs and monocytes cell culture. Incubating together with the suitable antibody for half an hour at 4°, the cells were washed with FACS buffer and passed through a BD LSR II (BD Biosciences, San Diego, CA). The cells were incubated with anti‐CD4 FITC PerCP for 1 hour, subsequently being fixed and permeabilized by a fixation/permeabilization kit (BD Biosciences). Then, the cells were stained with anti‐Foxp3 and analyzed by flow cytometry. A FlowJo software (Tree Star) was used to collect and analyze the data.

### 2.9 Histological analysis and immunohistochemistry

Collected from recipients, the grafted tracheal specimens were fixed with 10% buffered formalin and dehydrated before preparation of paraffin sections. Subsequently, serial 4 μm thick sections were prepared and subjected to hematoxylin/eosin staining for routine histology.

Luminal occlusion was evaluated by a blinded, independent reader and then measured by using an ImageJ software (NIH, Bethesda, MD). The percentage of airway obstruction was calculated using the following formula: the obliterated lumen area divided by the total lumen area × 100%. For quantitative analysis of luminal obliteration of tracheal grafts, 5 μm routine sections of tracheal were incubated overnight with α‐SMA antibody (rabbit anti‐mouse; BD Biosciences). A proper irrelevant IgG was used for guarantee that nonspecific binding effects were identified as a negative control. The intensity of α‐SMA staining was determined by an independent, blinded reader with an Image‐Pro Plus version 6.0 software (Media Cybernetics). The rate of α‐SMA expressed area inside the cartilage ring was determined likewise as it is calculated in luminal occlusion. Quantitative analysis of α‐SMA‐positive areas in tracheal grafts was performed.

### 2.10 Enzyme‐linked immunosorbent assays

Enzyme‐linked immunosorbent assays (ELISA) was performed as we described previously.^7^ The IL‐10 and TGF‐β levels in the medium were determined with antibody‐coated ELISA kit (R&D Systems) in accordance with commercial protocols. The optical density (OD) values were determined at 450 nm by a Multiskan spectrophotometer (Thermo, San Jose, CA).

### 2.11 MicroRNA transfection

The mimic of miR‐27a‐3p, control, or miR‐27a‐3p‐inhibitor (GenePharma, Shanghai, China) was transfected by using Lipofectamine RNAiMAX reagent under the manufacturer's protocol (GenePharma) at 50 nM at final concentration. The efficiency of transfection was measured by analyzing miR‐27a levels via RT‐qPCR. The pcDNA3.1‐sprouty2 recombinant plasmid (Sangon, Shanghai, China) was transfected into the DCs. The transfection reactions were conducted using the RFectplasmid DNA transfection reagent (Changzhou Baidai Biotechnology Co. Ltd, China) in Opti‐MEM prewarmed at 37°C.

### 2.12 Statistical analyses

Each experiment was repeated at least in thrice, and the mean values (mean ± SD) are presented. One‐way analysis of variance and least significant difference (LSD) were used to compare differences between multiple groups. Significance were considered when *P* < .05. Graph Pad Prism software (San Diego, CA) and IBM SPSS Statistics 23 (IBM Corp, Armonk, NY) were used for the statistics analysis.

## 3 RESULTS

### 3.1 MiR‐27a‐3p‐modified BM‐derived DCs suppressed BO in murine orthotopic tracheal transplantation

**FIGURE 1:**
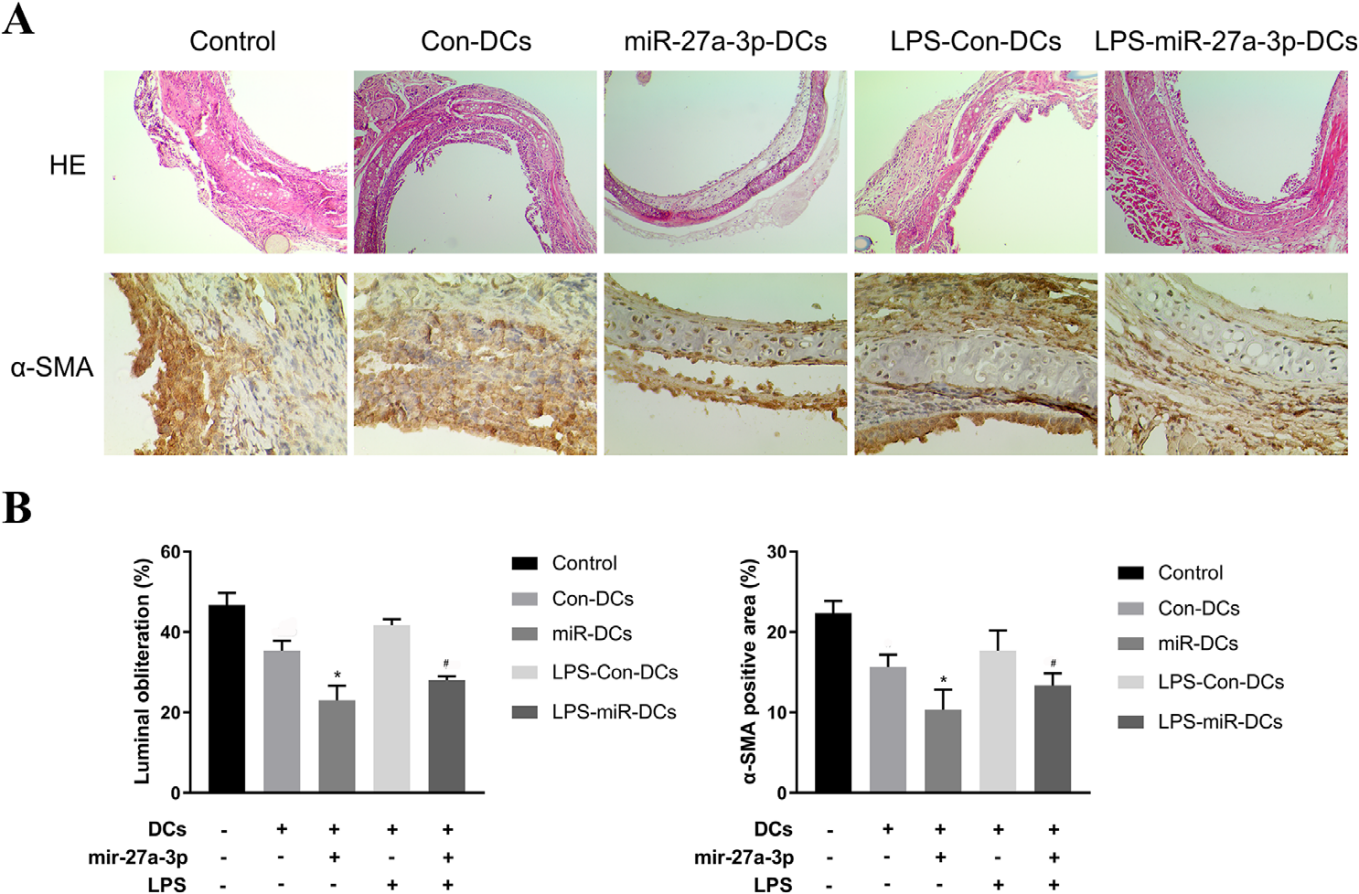
DCs were transfected with 50 nM control mimics (Con‐DCs), mimics for miR‐27a‐3p (mir‐27a‐3p‐DCs), LPS‐treated control mimics DCs (LPS‐Con‐DCs), and LPS‐treated miR‐27a‐3p transfected DCs (LPS‐mir‐27a‐3p‐DCs). Then, the grafts from recipients treated with PBS (Control) and other groups were collected. A, Histological features of syngeneic grafts and allografts on D30 after transplantation. Quantitive analysis of luminal obliteration of tracheal grafts (H & E; original magnification: ×100) and quantitive analysis of α‐SMA positive area in tracheal grafts (original magnification: ×400). B, The luminal obliteration of tracheal grafts and α‐SMA positive area of mir‐27a‐3p‐DCs group were significantly different compared with control and the Con‐DCs group. And the LPS‐miR‐27a‐3p‐DCs group was significantly different compared with LPS‐Con‐DCs group. n = 3; mean ± SD; *P< 0.05 for the difference compared with control and the Con‐DCs group; #*P*< 0.05 for the difference compared with the LPS‐Con‐DCs group

Our hypothesis is that miR‐27a‐3p‐modified immature dendritic cells (imDCs) would suppress BO. To confirm our hypothesis, we investigated luminal obliteration and α‐SMA expression on day 30 after transplantation in a murine model. The results showed that the luminal obliteration in the mir‐27a‐3p‐transfected DCs notably decreased compared with other groups. LPS are important factors that induce imDC maturation. Thus, the luminal obliteration in the LPS‐treated imDCs group was remarkably higher than in other groups; however, LPS‐treated mir‐27a‐3p‐imDCs still alleviated luminal obliteration. Myofibroblast marker α‐SMA expression was similar in all groups (Figure 1).

### 3.2 MiR‐27a‐3p upregulated the expression level of ERK and promoted IL‐10 secretion in DCs by targeting the ERK inhibitor, sprouty2

**FIGURE 2:**
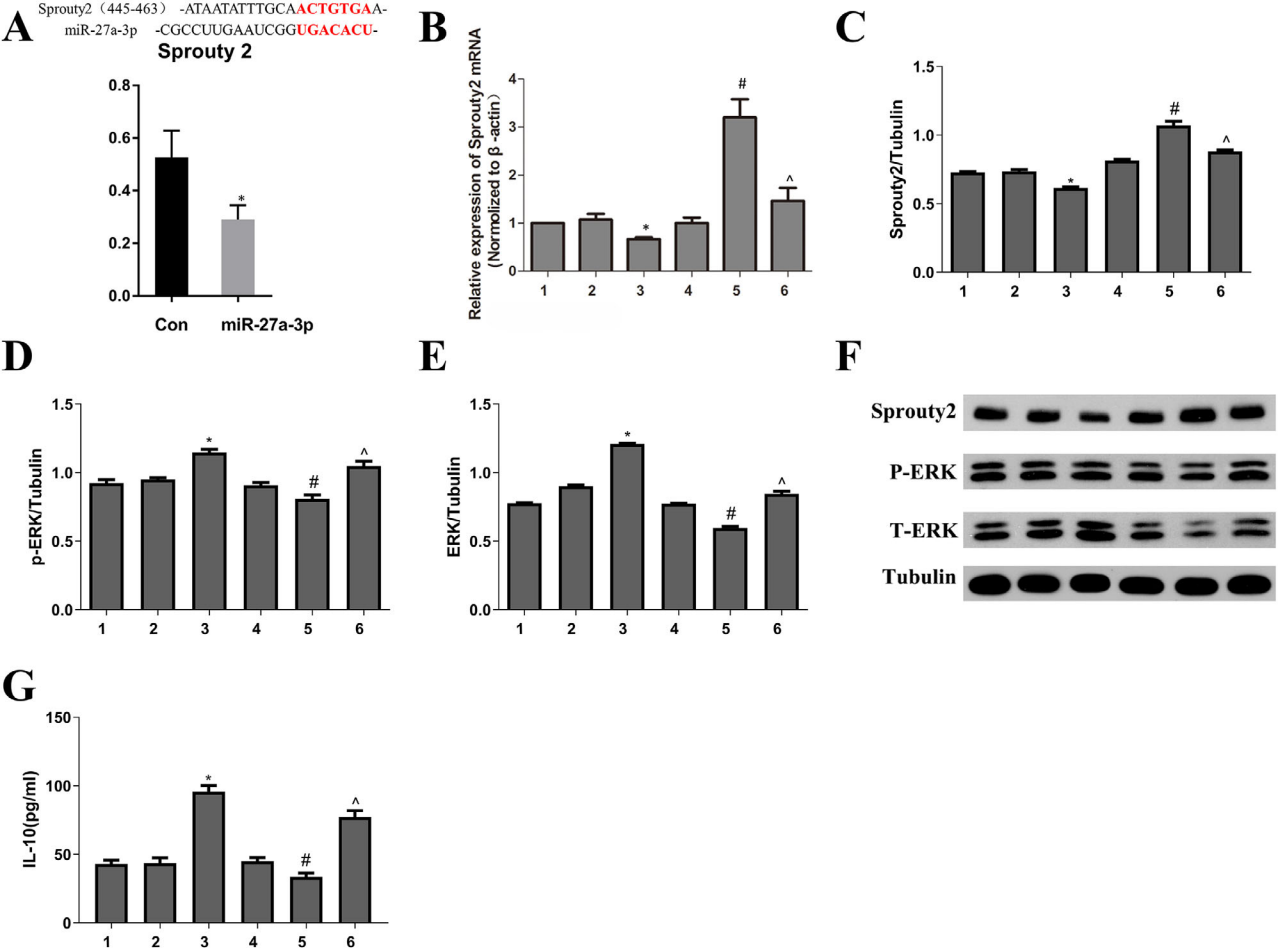
A, DNA fragments within the 3′UTRs of the *sprouty2* genes that contain the miR‐27a‐3p binding site were cloned into the luciferase reporter. Luciferase activity in the cells was measured. It confirmed that mir‐27a‐3p targets *Sprouty2* gene. B and C, Real‐time PCR and Western blot were used to observe Sprouty2 mRNA and protein expression. D‐F, Western blot was used to observe pERK and tERK protein expression. G, IL‐10 was examined by ELIAS. Data from three independent experiments; mean ± SD. ^*^ *P*< 0.05 for the difference compared with control and mimic NC group; ^#^ *P*< 0.05 for the difference compared with pcDNA3.1 group. ^*P*< 0.05 for the difference compared with the pcDNA3.1‐sprouty2 group. 1, control group; 2, mimics NC group; 3, mir‐27a‐3p mimics group; 4, pcDNA3.1 group; 5, pcDNA3.1‐sprouty2 group; 6, mir‐27a‐3p mimics‐pcDNA3.1‐sprouty2 group

We used TargetScan to predict the targets of miR‐27a‐3p. As a result, it could bind sequences within the 3′ UTRs of the *Sprouty2* gene. A luciferase reporter assay confirmed the prediction (Figure 2A). Expression of sprouty2 mRNA was remarkably downregulating in mir‐27a‐3p set than that in the control group or mimics‐NC groups. In addition, the sprouty2 mRNA expression in the mir‐27a‐3p‐pcDNA3.1‐sprouty2 group was significantly decreased (Figure 2B). The sprouty2 protein expression was in accordance with that of mRNA transcription level (Figure 2C). Sprouty2 is an inhibitor of ERK1/2. To investigate ERK activation, we measured the protein levels of total ERK (tERK) and phospho‐ERK (pERK). As it is shown in Figure 2D‐F, tERK and pERK increased in miR‐27a‐3p‐transfected DCs but decreased in PcDNA3.1‐sprouty2‐transfected DCs. Furthermore, we examined IL‐10 levels by ELISA; the results showed that IL‐10 production increased in miR‐27a‐3p‐transfected DCs (Figure 2G). These data suggest that mir‐27a‐3p targets sprouty2, indirectly increasing ERK activation and IL‐10 production in DCs.

### 3.3 MiR‐27a‐3p increased JAK1 and STAT3 expression by activating the JAK/STAT3 pathway

**FIGURE 3:**
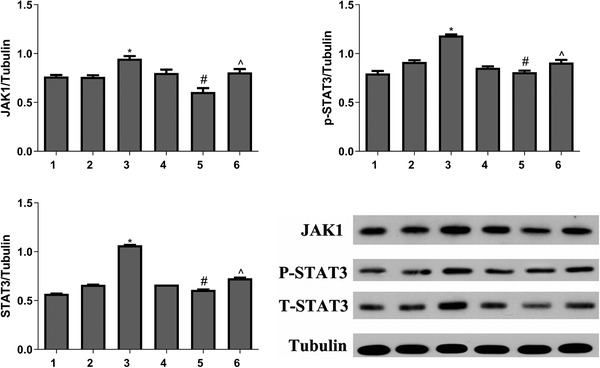
Protein levels of JAK1, p‐STAT3, and STAT3 were determined by Western blotting and analyses performed using ImageJ software. Data from three independent experiments; mean ± SD. *P< 0.05 for the difference compared with control and mimic NC group, #*P*< 0.05 for the difference compared with pcDNA3.1 group. ^*P*< 0.05 for the difference compared with pcDNA3.1‐sprouty2 group. 1, control group; 2, mimics NC group; 3, mir‐27a‐3p mimics group; 4, pcDNA3.1 group; 5, pcDNA3.1‐sprouty2 group; 6, mir‐27a‐3p mimics‐pcDNA3.1‐sprouty2 group

To investigate whether mir‐27a‐3p regulates JAK/STAT3 pathway activities, we examined its effect on the expression of JAK1, phospho‐STAT3 (p‐STAT3), and STAT3 proteins. As shown in Figure 3, the levels of all three proteins increased in miR‐27a‐3p‐transfected DCs but decreased in PcDNA3.1‐sprouty2‐transfected DCs.

### 3.4 MiR‐27a‐3p suppressed DC maturation

**FIGURE 4:**
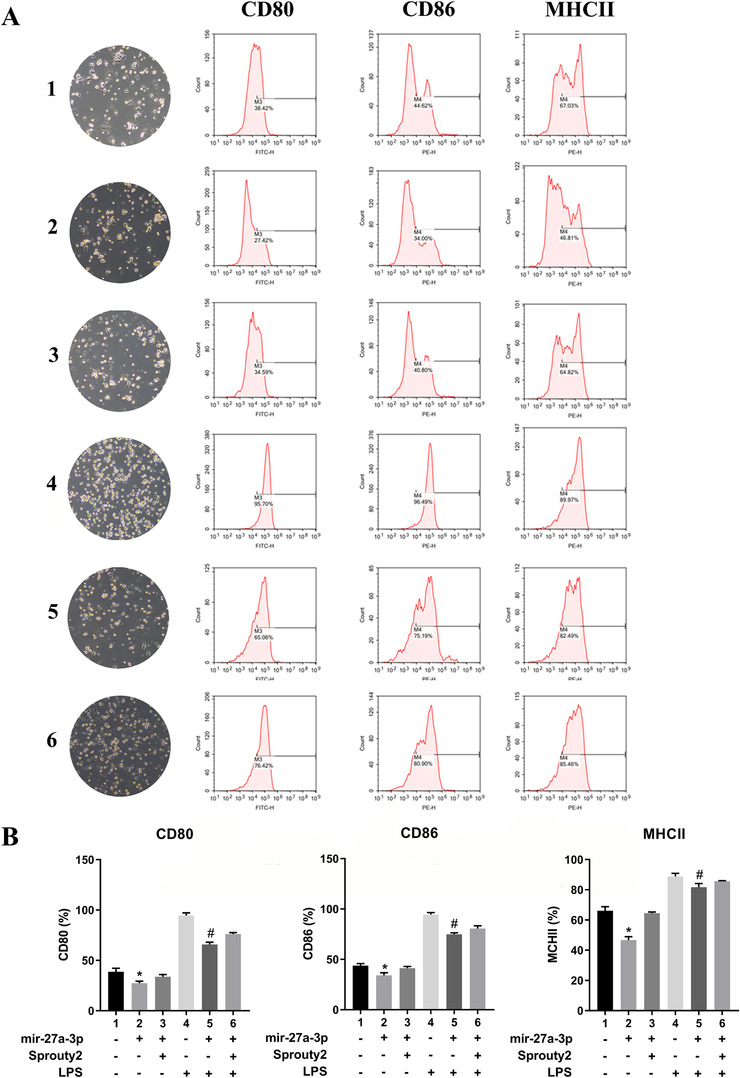
A, CD80, CD86, and MHCII were examined by flow cytometry and the morphology of cells was observed by a light microscope. (B) CD80, CD86, and MHCII expression data were analyzed. Data from three independent experiments; mean ± SD. *P< 0.05 for the difference compared with mimic NC group and mimics mir‐27a‐3p‐pcDNA3.1‐sprouty2 group, #*P*< 0.05 for the difference compared with LPS‐treated mimic NC group and mir‐27a‐3p mimics‐pcDNA3.1‐sprouty2 group. CD80, CD86, and MHCII expressions in LPS‐treated groups were significantly higher than in non‐LPS‐treated group. 1, mimics NC group; 2, mir‐27a‐3p mimics group; 3, mir‐27a‐3p mimics‐sprouty2 group; 4, LPS‐treated mimics NC group; 5, LPS‐treated mir‐27a‐3p mimics group; 6, LPS‐treated mir‐27a‐3p mimics‐pcDNA3.1‐sprouty2 group

To further explore the impacts of miR‐27a‐3p on DC maturation, the expressions of the DC maturation marker MHC‐II, CD86, and CD80 were determined. The mir‐27a‐3p‐transfected DC had lower MHC‐II, CD86, and CD80 level than the control group (mimics NC DCs) and miR‐27a‐3p‐Sprouty2 group. After stimulation for 12 h by LPS, MHC‐II, CD86, and CD80 expressions were prominently upregulated compared to the unstimulated groups (mimics NC DCs). Transfection of mir‐27a‐3p prominently downregulated the LPS‐induced upregulation of MHC‐II, CD86, and CD80. Moreover, transfected‐sprouty2 pretreatment partially offset the inhibition of miR‐27a‐3p on DC maturation (Figure 4).

### 3.5 Mir‐27a‐3p‐transfected DCs regulated Treg generation and cytokine production in vivo

**FIGURE 5:**
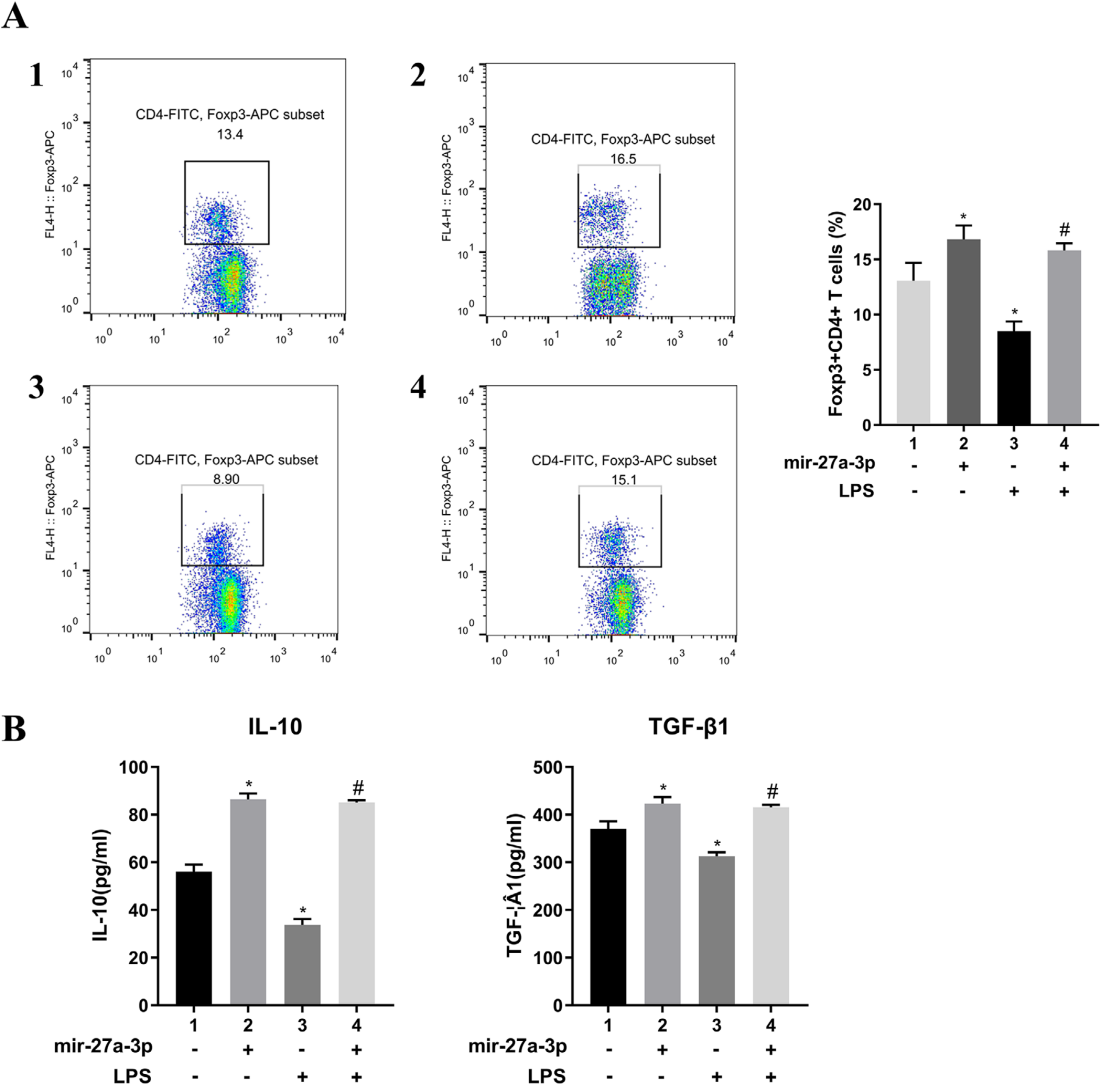
A, Foxp3^+^CD4^+^CD25^+^ T‐cell generation was examined by flow cytometry and in mimic mir‐27a‐3p group was significantly higher than in other groups. B, The cytokines IL‐10 and TGF‐β were examined by ELISA; results showed that the mimic mir‐27a‐3p group had elevated IL‐10 and TGF‐β, more so than other groups. Data from three independent experiments; mean ± SD. *P< 0.05 for the difference compared with mimic NC group. #*P*< 0.05 for the difference compared with LPS‐treated mimic NC group. 1, mimics NC; 2, mimics mir‐27a‐3p; 3, LPS + mimics NC; 4, LPS + mimics mir‐27a‐3p

Further studies examined the presence of Foxp3^+^CD4^+^ T cells using flow cytometry to explore the potential effect of mir‐27a‐3p‐transfected DCs on our murine model. As expected, Treg generation in mimic mir‐27a‐3p group was remarkably higher than that in mimic NC (control) and LPS‐treated groups (Figure 5A). Furthermore, the mir‐27a‐3p group more IL‐10 and TGF‐β comparing with the other groups and induced immune tolerance (Figure 5B).

### 3.6 Mir‐27a‐3p‐suppressed myofibroblast differentiation through TGF‐β/Smad pathway by targeting Smad2 and Smad4

**FIGURE 6:**
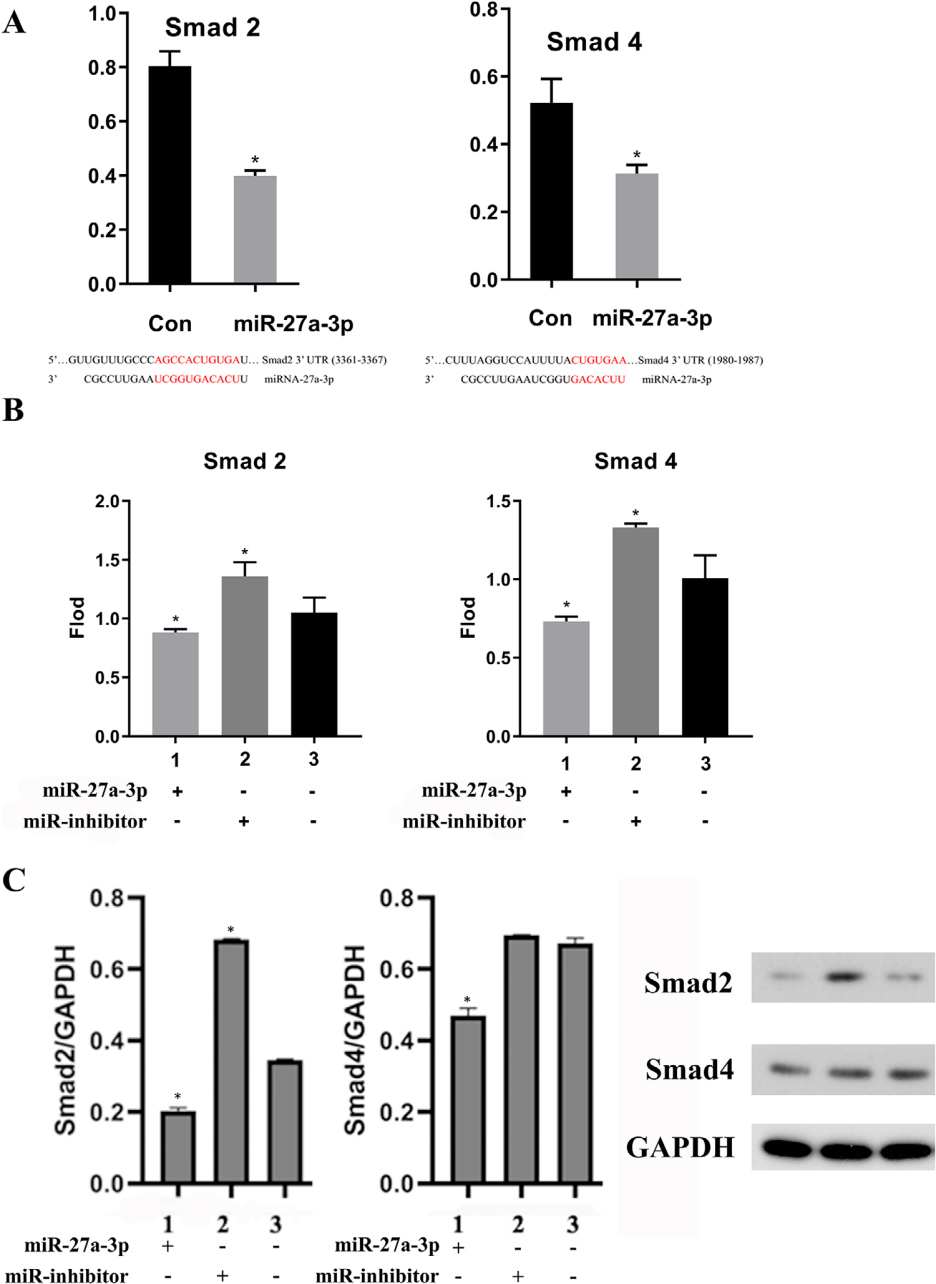
A, DNA fragments within the 3′UTRs of the *Smad2* and *Smad4* genes that contain the miR‐27a‐3p binding site were cloned into the luciferase reporter. Luciferase activity in the cells was measured. We used real‐time PCR to determine the RNA levels of Smad2 and Smad4 (B); protein levels were determined by Western blotting and analyses were performed using ImageJ software (C). Data from three independent experiments; mean ± SD. *P< 0.05 for the difference compared with mimic NC group. 1, mimic mir‐27a‐3p group; 2, mir‐27a‐3p inhibitor group; 3, mimic NC group

Mir‐27a‐3p‐induced TGF‐β overexpression may regulate the immune system without activating the TGF‐β/Smad pathway that induces myofibroblast differentiation. We used TargetScan to identify miR‐27a‐3p targets. The results indicated its binding sequences within the 3′ UTRs of the *Smad4* and *Smad2* genes. Next, we cloned the 3′ UTRs of these genes downstream of a luciferase reporter and transfected them with either control or miR‐27a‐3p mimics. This result illustrated that miR‐27a‐3p dramatically downregulated luciferase activities of reporters that contained the 3′ UTRs of Smad4 and Smad2 (Figure 6A), suggesting that miR‐27a‐3p directly targeted these genes. Next, we overexpressed and knocked down miR‐27a‐3p in NIH‐3T3 cell line to examineα‐SMA, Smad2, and Smad4 at the mRNA transcription level (Figure 6B) and protein level (Figure 6C). We observed that Smad2 and Smad4 presented a remarkable increase in miR‐27a‐3p inhibitor group while decrease in the miR‐27a‐3p group.

## 4 DISCUSSION

**FIGURE 7:**
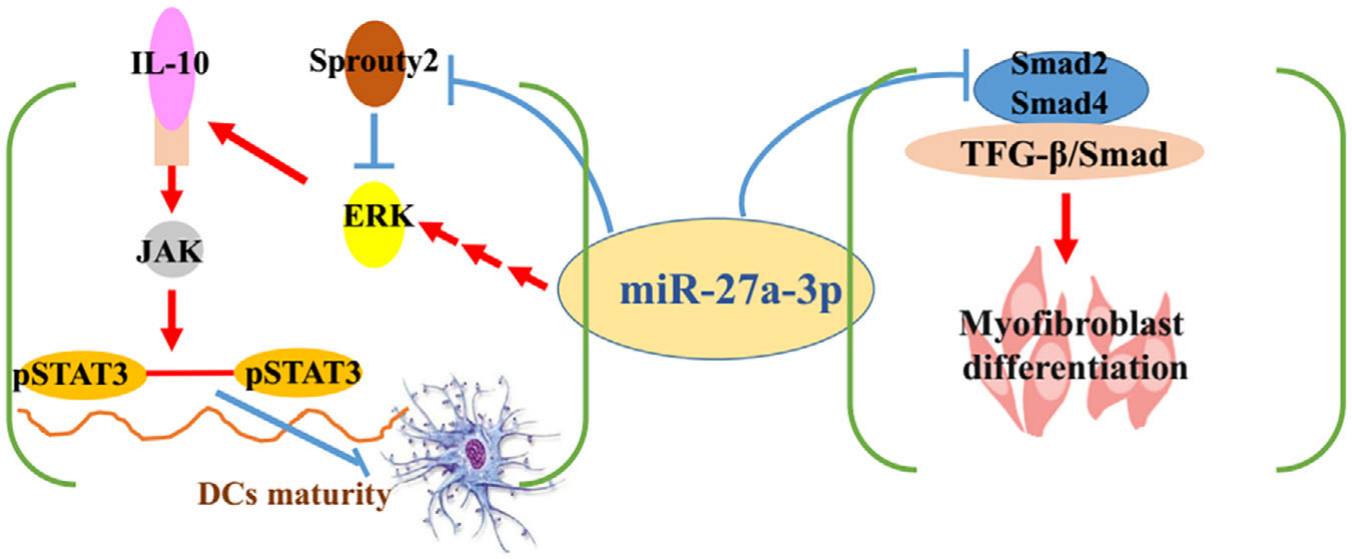
Mir‐27a‐3p‐transfected DCs suppress OB on murine orthotopic tracheal transplantation model via inducing immune tolerance and inhibiting the progression of myofibroblast differentiation

In the present research, we illustrated that mir‐27a‐3p‐transfected BM‐derived DCs could suppress BO in a rodent orthotopic tracheal transplantation model; the mechanism by which this occurs included two aspects (Figure 7). First, it involved the induction of immune tolerance; we illustrated that mir‐27a‐3p maintained the immature state of DCs through targeting sprouty2 that indirectly increased the expression of ERK, which in turn promoted IL‐10 production in DCs. IL‐10 is an important regulator of immunosuppression; it could activate the JAK/STAT3 signaling, regulate DC maturation, and induce the enrichment of CD4^+^ Foxp3^+^ Treg cells, subsequently increasing TGF‐β synthesis. The second part was the inhibitory effect of myofibroblast differentiation. Furthermore, mir‐27a‐3p also inhibited TGF‐β/Smad pathway and suppressed fibrosis by targeting Smad2 and Smad4.

As potent antigen‐presenting cells, DCs’ function includes two facets; they induce either tolerance or immunity. Tolerogenic DCs are resistant to maturation and are characterized by lower expression of CD80 or CD86, synthesis of anti‐inflammatory cytokines, such as IL‐10 and TGF‐β, and inhibits T‐cell proliferation. In contrast, immunogenic DCs strongly express CD80 and CD86 could synthesize pro‐inflammatory cytokines including TNFα, and have the ability to stimulate T‐cell proliferation.^16^ Therefore, regulation of DC maturation plays a pivotal role in Treg proliferation and tolerance induction. DC maturation is dependent on many factors and signaling pathways. STAT3 is a potential intrinsic negative regulator of DC maturation.^22^ Activation of STAT3 phosphorylation leads to suppression of CD80 and CD86 transcription, and the regulation of DC maturation.^26^ However, it is documented that Stat3‐deficient DCs could enhance immune activity, resist IL‐10‐mediated immunosuppression, and activate T cells.^27^ IL‐10/JAK/STAT3 is a well‐tested signaling cascade that can control the anti‐inflammatory response signal cascade.^21^ As one of the primary immunosuppressive cytokines, IL‐10 could be produced by almost of leukocytes, including macrophages, DCs, and B‐cell, and many CD4+ T‐cell cluster (including Tregs). ERK activation elicits high levels of IL‐10 from DCs.^28^, ^29^

miRNAs are crucial in the differentiation, survival, hematopoiesis, and immune functional cells, such as monocytes and DCs; miR‐27a‐3, serving as miR‐23∼27‐24 cluster member, has been shown to facilitate the Tr1 (CD4^+^IL‐10^+^) accumulation mediated by DCs and to increase IL‐10 secretion.^30^ Our study suggests that mir‐27‐3p promotes IL‐10 production in DCs via ERK signaling. However, the effect is indirect; evidently, mir‐27a‐3p lowered the level of the ERK inhibitor sprouty2 and consequently increased ERK activation (Figure 2).

IL‐10 overexpression maintains tolerance and immune homeostasis by activating the IL‐10/JAK/STAT3 cascade^31^; JAK/STAT3 pathway activation has been shown to suppress DCs activation.^32^, ^33^ In our study, mir‐27a‐3p elicited IL‐10 secretion and increased the levels of the JAK1/STAT3/pSTAT3 proteins (Figure 3). Treatment of DCs with LPS increased the levels of the surface markers CD80, CD86, and MHCII compared with mir‐27a‐3p‐DCs (Figure 4). Therefore, we illustrate that mir‐27a‐3p regulates DC maturation as well as function by first targeting sprouty2, which indirectly activates ERK and promotes IL‐10 and second, via the JAK1‐STAT3 pathway.

In a murine orthotopic tracheal transplantation model, miR‐27a‐3p‐modified DCs suppressed BO; luminal obliteration was less than in the other groups (Figure 1). Moreover, mir‐27a‐3p could elevate the synthesis of the immunosuppressive cytokines, such as IL‐10 and TGF‐β, along with Foxp3^+^CD4^+^ T cells (Figure 5). We therefore hypothesized that mir‐27a‐3p activates a feedback regulatory loop to limit immune pathology. It activates IL‐10/JAK/STAT3 signaling, regulates DC maturation, increases IL‐10 production, and induces immune tolerance.

In our previous study, imDCs attenuated obliterative bronchiolitis in trachea allograft rats and elevated immunosuppressive cytokines IL‐10 and TGF‐β1 synthesis.^24^ IL‐10 participates in immune regulation to prevent BO. However, it has been documented that TGF‐β1 could stimulate the differentitation of myofibroblast and result in matrix deposition, which characterizes BO.^8^ Receptor/TGF‐β biology is upregulated in the setting of BO and is involved in the mechanisms of BO.^34^ TGF‐β1 is also an immunosuppressive element in a series of diseases that favors the acquisition of tolerogenic properties by BM‐derived dendritic cells.^35^ Retinoic acid could reduce rejection in a TGF‐β‐dependent manner by promoting the differentiation of Tregs and inhibiting Th17 cells differentiation in heart transplantation.^36^ It seems that TGF‐β is double‐edged, inducing tolerance but stimulating myofibroblast trans‐differentiation. Our study discovered that mir‐27a‐3p inhibited the TGF‐β/Smad pathway and suppressed myofibroblast differentiation through targeting Smad2 as well as Smad4 (Figure 6). Furthermore, in parallel with our previous study, miR‐27a‐3p could prevent the process of EMT in lung epithelial cells.^7^ These results illustrated that mir‐27a‐3p might act as a protective effect on trachea transplantation by regulating DC maturation, enhancing Treg production and inhibiting myofibroblast differentiation. Of course, our orthotopic tracheal transplantation model has some limitations; it has a nonvascularized graft and simulates the large airways instead of the pulmonary environment. However, its practicality for immunological and fibroproliferative studies^37^ allows hypothesis testing and answering many scientific questions.

In conclusion, we confirmed that mir‐27a‐3p‐modified DCs attenuated airway obliteration in a murine orthotopic trachea transplantation model. Further analysis suggested that mir‐27a‐3p regulates DC maturation by targeting sprouty2, which indirectly activates ERK and promotes IL‐10 through the IL‐10/JAK1/STAT3 protein signaling. In result, it created a positive feedback loop that could maintain DCs in an immature state. In this series of processes, Treg differentiation was increased and immune tolerance was regulated. Mir‐27a‐3p also regulated TGF‐β function, inhibited the TGF‐β/Smad pathway, and suppressed myofibroblast differentiation by targeting Smad2 and Smad4. Additionally, the advantage of antigen‐specific immunosuppressive therapies is that they induce immune tolerance without affecting the beneficial immune response. Accordingly, we confirmed the regulatory role of mir‐27a‐3p in DCs, providing a feasible clinically applicable strategy for BO treatment after LT.

## CONFLICT OF INTEREST

The authors declare that they have no conflict of interest.

## AUTHOR CONTRIBUTES

Ming Dong, Xin Wang, and Tong Li wrote this manuscript. Jing Wang analyzed all data. Honglin Zhao and Yaqing Jing performed the animal experiments. Yi Liu and Jun Chen performed cell and molecule experiments. All authors read and approved the final manuscript.
