# CORRIGENDUM

**DOI:** 10.1002/ctm2.433

**Published:** 2021-06-28

In Dong et al.,^1^ the following error was published in the article byline.

Ming Dong, Xin Wang, Tong L1, Jing Wang, Yunwei Yang, Yi Liu, Yaqing Jing, Honglin Zhao, Jun Chen

The author name, ‘Tong L1’, was incorrect and should have read, ‘Tong Li’. The corrected article byline is below:

Ming Dong, Xin Wang, Tong Li, Jing Wang, Yunwei Yang, Yi Liu, Yaqing Jing, Honglin Zhao, Jun Chen

The online version of this article was corrected.

We apologise for this error.

## References

[1] Dong M , Wang X , Li T , et al. Mir‐27a‐3p attenuates bronchiolitis obliterans in vivo via the regulation of dendritic cells’ maturation and the suppression of myofibroblasts’ differentiation. Clin Transl Med. 2020;10:e140. 10.1002/ctm2.140. 32898329 PMC7423186

